# An enhanced adaptive non-local means algorithm for Rician noise reduction in magnetic resonance brain images

**DOI:** 10.1186/s12880-019-0407-4

**Published:** 2020-01-06

**Authors:** Kaixin Chen, Xiao Lin, Xing Hu, Jiayao Wang, Han Zhong, Linhua Jiang

**Affiliations:** 1 Shanghai Key Lab of Modern Optical Systems, School of Optical-Electrical and Computer Engineering, University of Shanghai for Science and Technology, 200093, P, Shanghai, R China; 2 School of Medicine, Stanford University, 269 Campus Drive, Stanford, CA 94305 USA

**Keywords:** MR brain images, Rician noise reduction, Median absolute deviation (MAD), Adaptive non-local means (NLM)

## Abstract

**Background:**

The Rician noise formed in magnetic resonance (MR) imaging greatly reduced the accuracy and reliability of subsequent analysis, and most of the existing denoising methods are suitable for Gaussian noise rather than Rician noise. Aiming to solve this problem, we proposed fuzzy c-means and adaptive non-local means (FANLM), which combined the adaptive non-local means (NLM) with fuzzy c-means (FCM), as a novel method to reduce noise in the study.

**Method:**

The algorithm chose the optimal size of search window automatically based on the noise variance which was estimated by the improved estimator of the median absolute deviation (MAD) for Rician noise. Meanwhile, it solved the problem that the traditional NLM algorithm had to use a fixed size of search window. Considering the distribution characteristics for each pixel, we designed three types of search window sizes as large, medium and small instead of using a fixed size. In addition, the combination with the FCM algorithm helped to achieve better denoising effect since the improved the FCM algorithm divided the membership degrees of images and introduced the morphological reconstruction to preserve the image details.

**Results:**

The experimental results showed that the proposed algorithm (FANLM) can effectively remove the noise. Moreover, it had the highest peak signal-noise ratio (PSNR) and structural similarity (SSIM), compared with other three methods: non-local means (NLM), linear minimum mean square error (LMMSE) and undecimated wavelet transform (UWT). Using the FANLM method, the image details can be well preserved with the noise being mostly removed.

**Conclusion:**

Compared with the traditional denoising methods, the experimental results showed that the proposed approach effectively suppressed the noise and the edge details were well retained. However, the FANLM method took an average of 13 s throughout the experiment, and its computational cost was not the shortest. Addressing these can be part of our future research.

## Background

In recent years, magnetic resonance imaging, as a commonly used medical imaging technology, has played an important role in medical diagnosis and brings great convenience to medical workers. The accuracy of clinical diagnosis is dependent on the quality of the MR image, but the noise generated during the imaging process reduces the quality of the MR image. So the noise made the boundaries of some tissue structures blurred [1], which resulted in increased the difficulty in recognizing the image details. Therefore, MR images denoising is a crucial step.

Currently there are many different denoising methods, for example the wavelet transform [2–5], anisotropic diffusion filter (ADF) [6, 7], linear minimum mean square error (LMMSE) [8, 9] and the maximum likelihood approach [10, 11]. However, most of the denoising algorithms are only suitable for Gaussian noise, and ignored the influence of Rician noise in MR images. Recently, the non-local means algorithm (NLM) proposed by Buades et al. [12] utilized the redundancy of the image and takes its weighted average to reduce the noise. The NLM algorithm was designed to remove Gaussian noise, but when it was applied to remove the noise in MR images, some researchers found the NLM algorithm was better than the Gaussian filter, Wiener filter and other traditional filters. In order to speed-up the NLM filter, Coupe et al. [13] proposed block implementation and parallel computation to optimize the filter and reduce the calculation cost. Thaipanich et al. [14] used singular value decomposition to classify images into blocks, and adjusted the window size adaptively based on the block classification results. However, both methods were based on the assumption that the MR image noise was modeled as the Gaussian distribution, and were not suitable for Rician noise. A new method is needed for MR images with Rician noise.

In this paper, we proposed an adaptive NLM approach for the distribution of Rician noise: this approach is called FANLM. As we mentioned above, the proposed methods in [13, 14] were based on the assumption that the noise is modeled as the Gaussian distribution. To get accurate results, the proposed method used the improved MAD estimator for Rician noise to estimate the characteristics of each pixel in the MR images, and selected the optimal search window size according to the estimated noise variance. Moreover, in order to better preserve the details, the fuzzy c-means approach was used before denoising to divide the membership degree of MR image. Additionally, we used the improved adaptive NLM algorithm for denoising which designed three window size as small, medium, and large, and selected the optimal search window size for each pixel by the improved MAD estimator for Rician noise.

In order to verify the superiority of the proposed approach, we experimented with simulating human brain MR image data and real human brain MR data, and compared the proposed approach with the traditional non-local means (NLM), linear minimum mean square error (LMMSE) and undecimated wavelet transform (UWT). The experimental results showed that the proposed method FANLM can better preserve the image details and remove noise, and the computational cost was also relatively reduced.

## Method

### Brief review of non-local means

The NLM filter exploits the data redundant among the patches in noisy images. In the filtered images, each pixel is a weighted average of non-local pixels. The mathematical expression of image noise can be expressed as *v*(*i*) = *u*(*i*) + *η*(*i*), where *v*(*i*) and *u*(*i*) are the noisy and noise-free image pixels at pixel *i*, respectively, where *η*(*i*) is a noise sample. By setting a search area, the estimated pixel value ${\hat{u}}_{NLM}(i)$ can be obtained by calculating the weighted average of the pixel values in the entire image, and is defined as follows:

1 $$ {\hat{u}}_{NLM}(i)=\sum \limits_{j\in {S}_i}w\left(i,j\right)v(j) $$

where the weight value *w*(*i*, *j*) is determined by the similarity of the pixels *i* and *j* in the search domain *S*_*i*_. The weights are defined as:

2 $$ w\left(i,j\right)=\frac{1}{z(i)}{e}^{-\frac{{\left\Vert p\left({N}_i\right)-p\left({N}_j\right)\right\Vert}_{2,a}^2}{h^2}} $$

Where,

3 $$ z(i)=\sum \limits_j{e}^{-\frac{d\left(i,j\right)}{h^2}} $$

In Eq. (2), *p*(*N*_*i*_) and *p*(*N*_*j*_) are the patches centered on pixel *i* and *j*. ${\left\Vert p\left({N}_i\right)-p\left({N}_j\right)\right\Vert}_{2,a}^2$ is the Euclidean distance between two patches weighted by a standard deviation *a* of the Gaussian kernel. The smoothing parameter *h* controls the smoothness of noise, and *z*(*i*) is the normalized factor.

B. Kang et al. [15] and h. Bhujle et al. [16] both adapted the local edge structures by changing the weight function. The difference is that B. Kang et al. proposed the expression of attenuation coefficient as a function of edge degree [15], which resulted in a modified weight function. They did not use the regular Euclidean distance in the calculation of weight function and calculated the distance between blocks based on the edge graph. Therefore, the weight function had changed automatically along with the calculation of similar distances. If the filtering parameters of the NLM method, e.g. the search window, the sliding window size, and the pixel weight, are fixed in the experiment, the denoising result will not be ideal, and image edges also won not be well retained. Therefore, we proposed a method that adaptively selected the filtering parameters based on noise variations.

### Brief review of fuzzy c-means

The fuzzy c-means (FCM) was proposed [17] since the traditional clustering method cannot divide image memberships well. The basic idea is to divide N vectors *x*_*i*_(*i* = 1, 2, 3…N) into C fuzzy groups and then determine the clustering center of each class, so as to minimize the similarity between different classes. The FCM algorithm is based on the minimization of the following objective functions:

4 $$ {J}_{FCM}=\sum \limits_{i=1}^C\sum \limits_{j=1}^N{\mu}_{ij}^m{\left\Vert {x}_j-{k}_i\right\Vert}^2 $$

For the image *I* = {*x*_*i*_ ∈ *I*| *i* = 1, 2, 3…*N*}, images with N pixels are divided into class C using the FCM algorithm. *μ*_*ij*_ is the value of membership of *ith* pixel in the cluster *j*. The clustering center *k*_*i*_ and fuzzy membership *μ*_*ij*_ are expressed as:

5 $$ {k}_i=\frac{\sum_{j=1}^N{\mu}_{ij}^m{x}_i}{\sum_{j=1}^N{\mu}_{ij}^m} $$

and

6 $$ {\mu}_{ij}=\frac{1}{\sum_{q=1}^c{\left(\frac{\left\Vert {x}_j-{k}_i\right\Vert }{\left\Vert {x}_j-{k}_q\right\Vert}\right)}^{2/\left(m-1\right)}} $$

satisfying the constraint conditions ${\sum}_{i=1}^C{\mu}_{ij}=1$.

However, FCM has its limitations: it can only segment simple texture images and is sensitive to noise; Thus, it cannot work well for noisy images. To solve this problem, s. Krinidis et al. [18] proposed a local fuzzy clustering algorithm (FLICM), but FLICM cannot make good use of the context information in the image for different local information. Gong, M et al. [19] and Maoguo, G et al. [20] made some improvements to FLICM, but the computational cost was still high.

Tao Lei et al. [21] put forward an improved robust FCM algorithm (FRFCM) which required less computational cost and was immune to noise. The FRFCM adopted a new approach that clustered the gray histogram of morphological reconstruction, and the membership degree of pixels were obtained through iteration. The FRFCM used the Lagrange multiplier to convert optimization problems into unconstrained optimization problems. The objective functions are as follows:

7 $$ {J}_M=\sum \limits_{i=1}^C\sum \limits_{j=1}^N{\mu}_{ij}^m{\left\Vert {x}_j-{v}_i\right\Vert}^2-\lambda \left(\sum \limits_{j=1}^N{\mu}_{ij}-1\right) $$

The main improvements of the FRFCM were two-fold. First, morphological reconstruction was introduced into FCM to smooth the image, which improved the robustness of the algorithm and preserved the details of the image. Second, the FRFCM modified the classification of membership by using a faster filter instead of the slower distance computation between pixels in local spatial neighbors and clustering centers.

## Proposed method

The traditional NLM denoising algorithm used a fixed size search window for each pixel. However, image pixels may be located in smooth or non-smooth areas. Suppose the pixel is in a non-smooth or textured area and the size of search window is still big, then denoising area will become more blurred due to the mean calculation; Similarly, if the smoothed area is filtered with a small search window, the variance of the filtered area will be much different from that of the original image. Therefore, we need to take into consideration the regional characteristics of each pixel and select the best size of search window based on the estimated variance of the search region. The proposed method selected the best window size adaptively: small(s), medium(m), large(l). To accurately obtain the image features of each region, we need to estimate the noisy image to obtain the noise variance. Our proposed FANLM method is to estimate the noise variance by the improved MAD estimator for Rician noise. In order to better preserve the details, we used FRFCM to cluster brain MR images to determine pixels membership in the region of interest. Then, the FANLM denoised the brain MR image based on the estimated image variance and adaptively selected optimal search window size.

### Estimation of Rician noise

The noise can be estimated by using methods based on wavelet transform [22] or principal component analysis (PCA) [23]. The method based on PCA is suitable for weak texture images but not so good for Rician noise estimation. In the wavelet transform domain, we need to filter the image in the horizontal and vertical directions respectively to achieve wavelet multi-resolution decomposition. The divided sub-bands include LL, HL, LH and HH sub-bands, where the HH sub-band is composed of the wavelet noise coefficient which generated after convolution [24]. So the median absolute deviation estimator used the wavelet coefficients of the HH sub-band to estimate the image noise. The estimated standard deviation expression is as follows:

8 $$ \hat{\sigma}=\frac{median\left(\left| HH\right|\right)}{0.6745} $$

However, the noise estimation method is suitable for Gaussian noise. An improvement of estimating Rician noise is proposed by Guan, K et.al [25].. The main improvement depended on a fixed-point formula for the signal-to-noise ratio and the correction factor ζ. And the variance of the amplitude image can be expressed as:

9 $$ {\sigma}_n^2=\upzeta \left(\theta \right){\hat{\sigma}}^2 $$

Where θ ≡ SNR, $\hat{\sigma}$ is estimated by the MAD for the initialization process and also provides a correction factor ζ:

10 $$ \upzeta \left(\theta \right)=2+{\theta}^2-\frac{\pi }{8}\mathit{\exp}\left(-\frac{\theta^2}{2}\right){\left(\left(2+{\theta}^2\right){I}_0\left(\frac{\theta^2}{4}\right)+{\theta}^2{I}_1\left(\frac{\theta^2}{4}\right)\right)}^2 $$

*I*_1_is the first-order modified Bessel function that iterated through the correction factor until it converges, or reached a given number. The iteration would converge When |*θ*_*i*_ − *θ*_*i* − 1_| ≤ *ε* , ε = 1.0 × 10^−8^, and the iterative correction scheme can be expressed as:

11 $$ {\theta}_i=\sqrt{\upzeta \left({\theta}_{i-1}\right)\left(1+\frac{{\overline{m}}_0}{{\hat{\sigma}}^2}\right)-2} $$

Where ${\overline{m}}_0$ is the average signal of the data, and $\hat{\sigma}$ is the average noise difference calculated by MAD estimator.

### Adaptive NLM to Rician noise

The basic idea of this section is to obtain the optimal size of the search window according to the variance of original images and filtered images. With the traditional NLM algorithm, the noisy images are represented as ${f}_i^s$, ${f}_i^m$ and ${f}_i^l$ respectively, and the corresponding variance of each region is expressed as ${\sigma}_{f,s}^2$, ${\sigma}_{f,m}^2$ and ${\sigma}_{f,l}^2$. The estimated variance ${\hat{\sigma}}_n^2$ was obtained by using the improved MDA method for the Rician noise, and then the estimated variance of the original image can be expressed as:

12 $$ {\hat{\sigma}}_{g,c}^2=\mathit{\max}\left({\sigma}_{f,c}^2-{\hat{\sigma}}_n^2,0\right) $$

Where *c* ∈ {*s*, *m*, *l*}. We used the traditional NLM algorithm to filter the image. The filtered images obtained by small, medium and large search window are denoted as ${\hat{u}}_{NLM}^s$, ${\hat{u}}_{NLM}^m$ and ${\hat{u}}_{NLM}^l$. Then we can estimate the noise variance of the filtered image using the improved MAD estimator for Rician noise. The variances in the small, medium and large regions are expressed as ${\hat{\sigma}}_{NLM,s}^2$, ${\hat{\sigma}}_{NLM,m}^2$ and ${\hat{\sigma}}_{NLM,l}^2$. The optimal search window size is expressed by the following expression:

13 $$ size\left({S}_i^{best}\right)={argmin}_c\left\{\left|{\hat{\sigma}}_{g,c}^2-{\hat{\sigma}}_{NLM,c}^2\right|\right\} $$

In order to further improve the quality of the filtered image, we added FCM as a subsequent processing step. Traditional FCM is sensitive to noise and cannot remove noise very well. We used the improved and robust FCM algorithm (FRFCM) proposed by Tao Lei et al. [20]. The FRFCM algorithm used local membership filter instead of filtering the distance between the pixel and cluster center in traditional FCM algorithm, and it was able to speed-up the FCM algorithm. At the same time, the morphological reconstruction was used to smooth the image, and the processed image edge details were enhanced.

### Datasets

#### Dataset 1

**Fig. 1:**
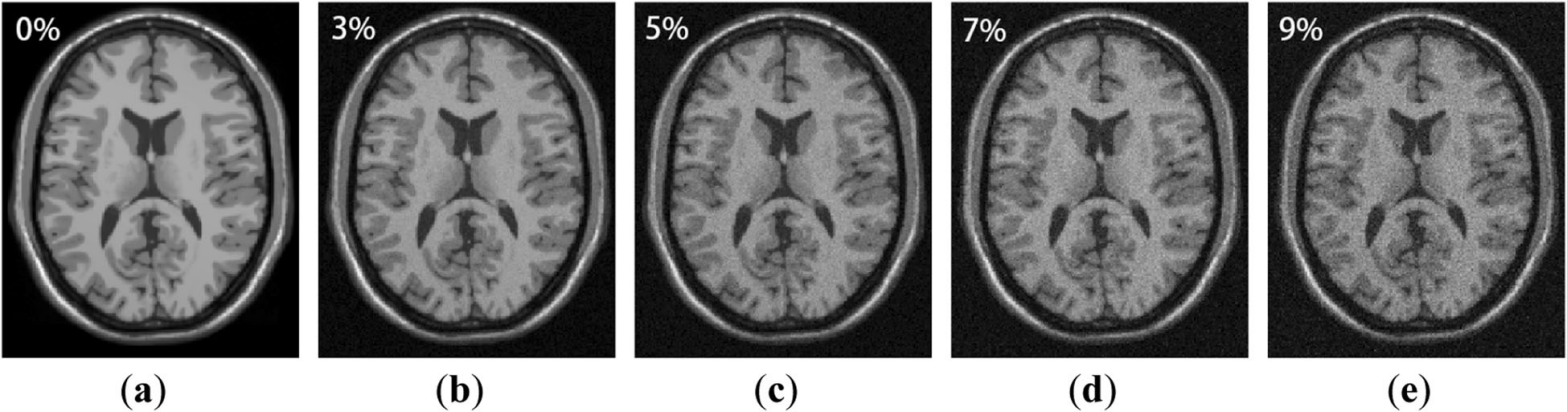
Brain MR noisy images with 0, 3, 5, 7, 9% noise level

The experimental data in this part was downloaded from the BrainWeb [26]. We downloaded the T1-weighted brain MR images which are corrupted with 0, 3, 5, 7, and 9% of Rician noise, and the MR image size is 181 × 217 × 181 voxels with 1 mm slice thickness. Different level of noisy MR images are shown in Fig. 1.

**Fig. 2:**
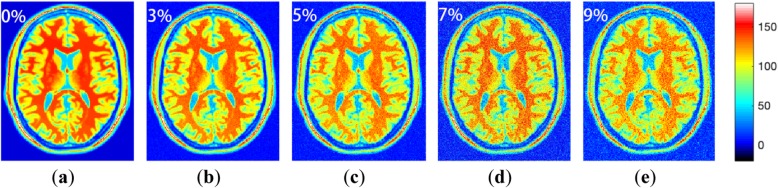
Colored Brain MR noisy images with 0, 3, 5, 7, 9% noise level

Since the naked eye cannot distinguish the difference of noise levels very well, we used colormap to dye the images. As shown in Fig. 2, we can clearly see that as the noise level increases, the boundaries of the MR images became more and more blurred, and some structural details have been lost. So it is necessary to remove the noise in MR images.

#### Dataset 2

**Fig. 3:**
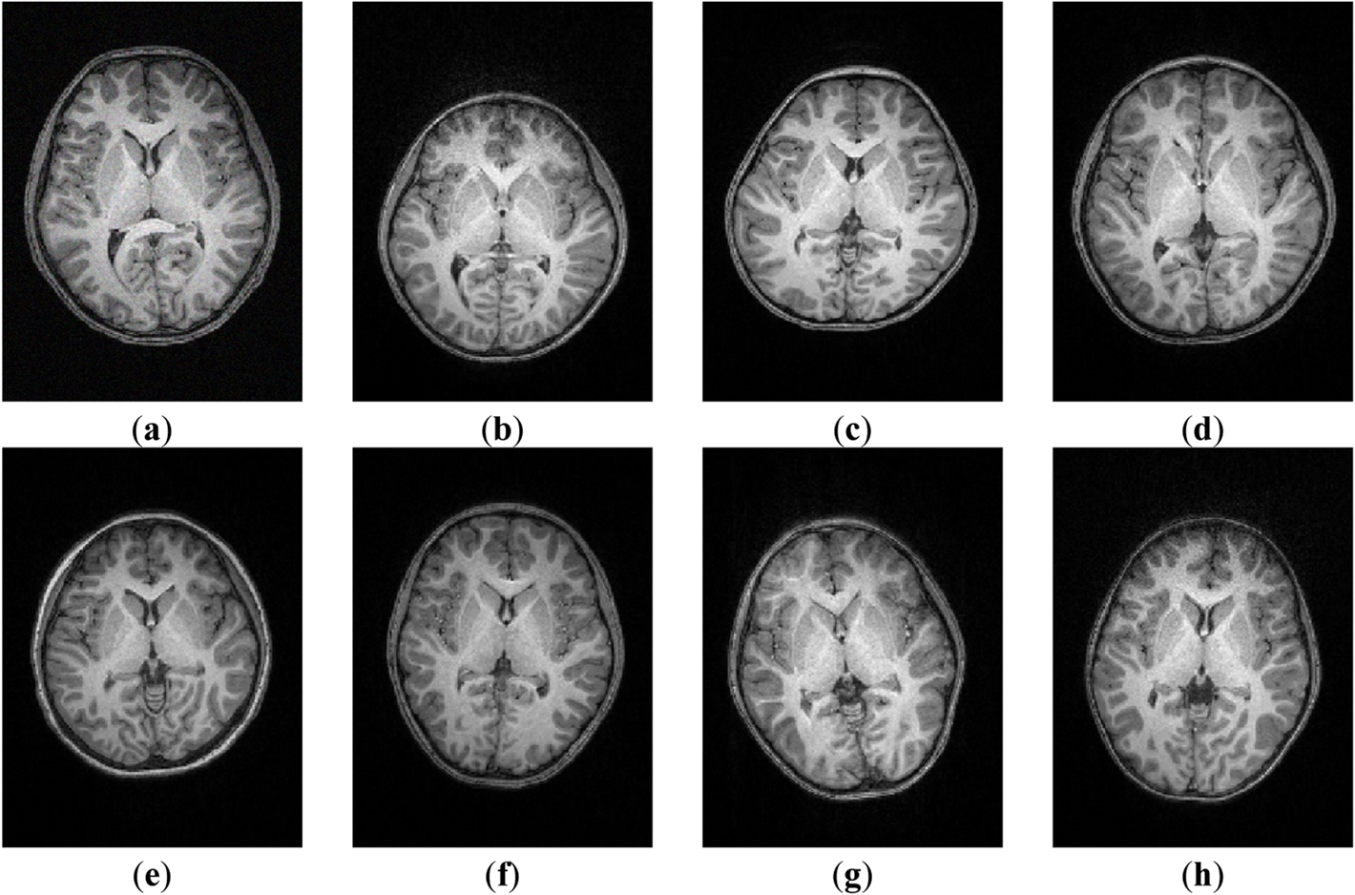
Real human brain MR images

The dataset 2 is real human brain MR images collected by using the Siemens MAGNETOM Prisma 3 T magnetic resonance imaging system in the State Key Laboratory of Cognitive Neuroscience and Learning at Beijing Normal University. The data consisted of 8 T1-weighted brain MR images as shown in Fig. 3, and the size of images is 192 × 256 × 170 with resolution of 1 mm.

### Selecting size of search window

Selection of the search window size through experiments have been discussed in some articles. The experiment in Buades’s study [27] showed that we can use 7 × 7 or 9 × 9 (window size) when processing gray images. And for color images with less noise, we can use 3 × 3 or 5 × 5 window size. Some researchers [28, 29] suggest that square search window in the range of 9 × 9 to 21 × 21 can be used for optimal algorithm and better experimental results. We found that it was difficult to remove noise by using a smaller window in our experiments. Therefore, we designed different sizes of the search window when processing different noisy images. In our proposed NLM approach, we chose 7 × 7 for small window, 13 × 13 for medium window, and 21 × 21 for large window.

## Results

In our study, four methods were used to denoise the noisy MR images: the proposed fuzzy c-means and adaptive non-local means (FANLM), non-local means (NLM), linear minimum mean square error (LMMSE), undecimated wavelet transform (UWT). All the experiments were conducted in MATLAB on Intel(R) Core i5–3470, 3.40Ghz CPU with 4GB RAM.

To evaluate and analyze the quality of the denoised MR images, two criteria were used: the structural similarity (SSIM) and the peak signal-to-noise ratio (PSNR). The experiment results were divided into two parts, one is the analysis result of the MR images downloaded from BrainWeb with noise levels of 3, 5, 7 and 9% respectively, and the other is the real data images acquired by MAGNETOM Prisma.

### Dataset 1

**Fig. 4:**
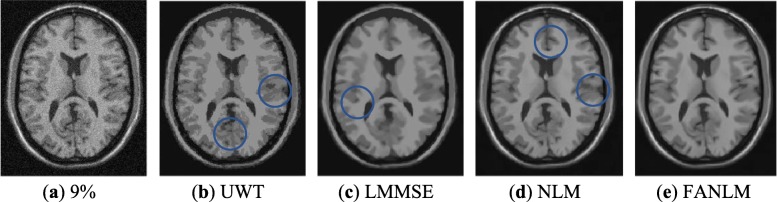
Experimental results of different methods. (**a**) corrupted MR image with 9% Rician noise; (**b**) UWT; (**c**) LMMSE; (**d**) NLM; (**e**) FANLM

We denoised the MR images with noise levels of 3, 5, 7 and 9% by using the proposed FANLM method and the NLM, LMMSE and UWT respectively. As shown in Fig. 4, it displays the original MR image (noise level of 9%) and the denoise results obtained by the four methods.

As shown in Fig. 4, compared with the FANLM, the UWT removed most of the noise, but the details were also removed in the meantime, and the structural details were not well preserved. The problems of LMMSE are that the edge details were not preserved as well, and ringing artifacts occurred in some parts of MR images. NLM used the fixed parameters and the same size of search window to denoise different regions, which resulted in excessive smoothing in some texture regions. However, the proposed method FANLM method was able to preserve structural details effectively. In order to see the difference in denoising more clearly, we circled these different places in the image.

**Fig. 5:**
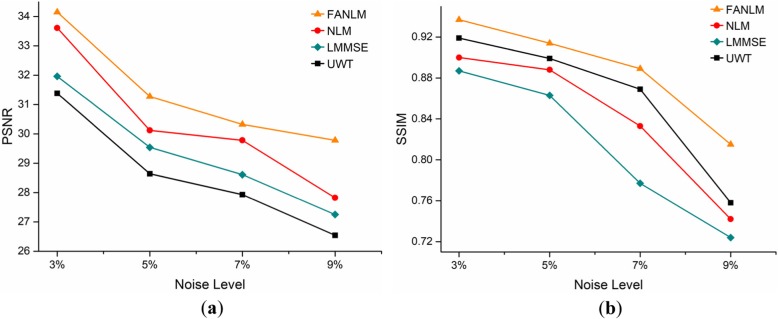
Comparison of the PSNR and SSIM of four methods. (**a**) PSNR; (**b**) SSIM

The above analysis of denoising results are only compared to the perspective of vision. In order to further verify the effectiveness of the proposed algorithm, the Fig. 5 shows the PSNR and SSIM obtained by the four methods when the original image had 3, 5, 7, and 9% R Rician noise levels. The results showed that our proposed method FANLM achieved the optimal PSNR and SSIM at different noise levels.

### Dataset2

**Fig. 6:**
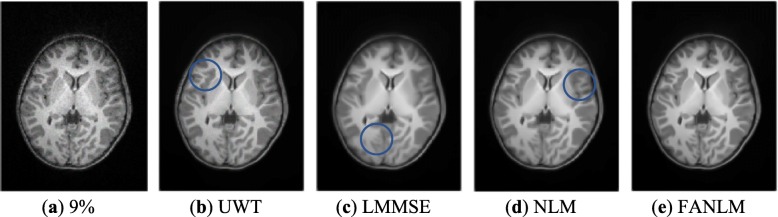
Experimental results of different methods. (**a**) corrupted MR image with 9% Rician noise; (**b**) UWT; (**c**) LMMSE; (**d**) NLM; (**e**) FANLM

**Fig. 7:**
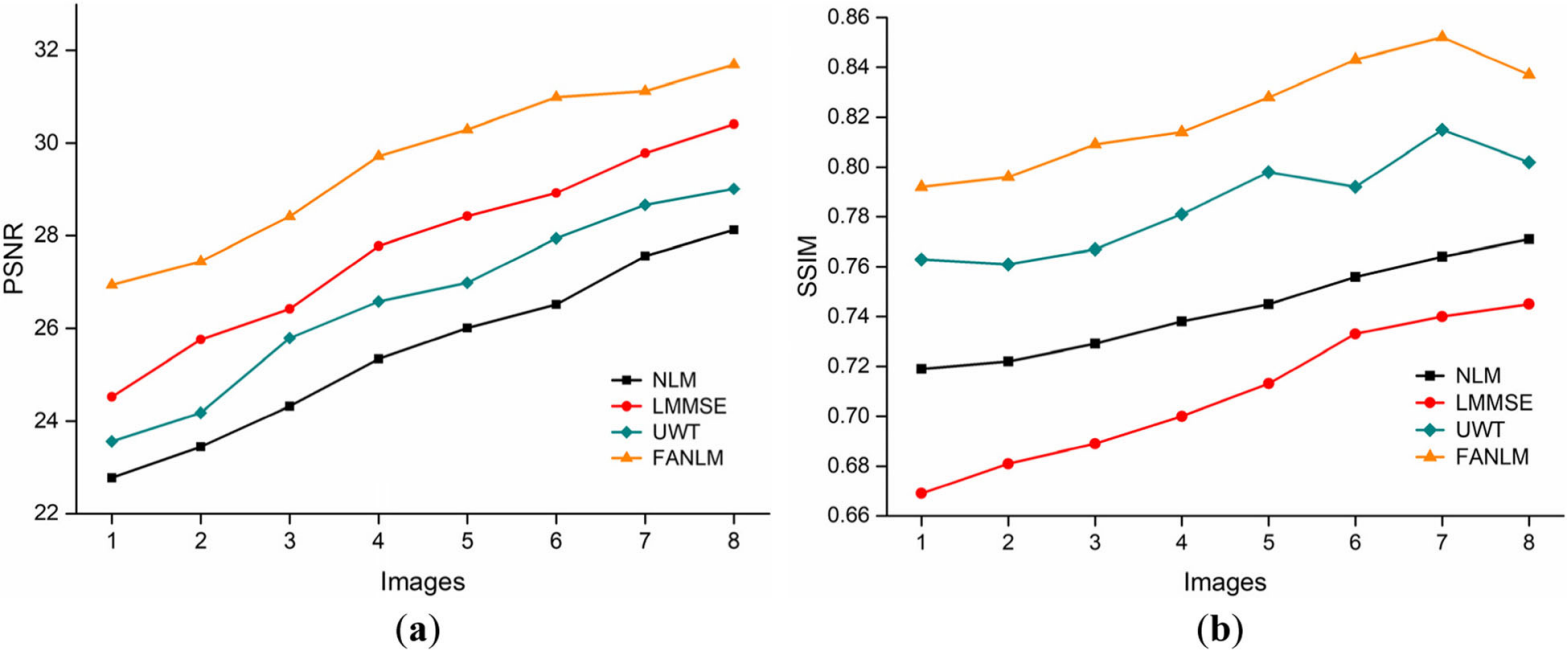
Comparison of the PSNR and SSIM of four methods. (**a**) PSNR; (**b**) SSIM

In this part, the real brain MR images were filtered by the NLM, LMMSE and UWT and FANLM method. Figure 6 presents the results in terms of visual quality, and Fig. 7 lists the PSNR and SSIM values.

We cannot intuitively tell the denoising performance difference based on the denoised image. But if we carefully observe and compare the edge details of the denoised image, we find that FANLM can better retain the edges and while suppressing noise well.

Figure 7 shows the PSNR and SSIM values of eight real brain MR images when using the four different methods. The results show that our proposed method achieved the highest PSNR and SSIM, which means that our method is superior to the other approaches.

## Discussion

Quantitative results are compared in the above section, and the results show that the proposed method can not only remove the noise effectively but also well preserve the detailed structures. However, the computational cost of the proposed method FANLM is only superior to that of NLM. In the future, we will make efforts to decrease computational complexity.

In Table 1, we list the computational times on datasets 1 for the four methods and with 3, 5, 7, and 9% Rician noise levels. In terms of PSNR and SSIM, the proposed FANLM in this paper is better than other methods. However, in terms of running time, it is obvious that the computational cost of this method is still not superior due to its complexity of calculating the weight and adaptively choosing the optimal window size. Although the computation time of FANLM is shorter than that of NLM, it is still longer than those of LMMSE and UWT. In the future, we will make efforts to decrease the computational complexity.

**Table 1:** Comparison of computational time on datasets 1

Denoising methods	Computational time (s)			
	3%	5%	7%	9%
FANLM	5.85	6.21	8.14	9.43
NLM	9.52	11.92	12.67	14.39
LMMSE	2.12	2.88	3.48	4.92
UWT	2.78	3.56	4.32	5.14

We also compared the computational cost on dataset 2, and the same problem existed. In Table 2, we list the computational costs of eight MR images. As stated above, the FANLM takes longer to calculate and has a computational burden due to its complexity of selecting the window size. Therefore, we need to improve the computation time in future works.

**Table 2:** Comparison of computational time on dataset 2

Denoising method	Computational time (s)							
	Image 1	Image 2	Image 3	Image 4	Image 5	Image 6	Image 7	Image 8
FANLM	16.93	17.35	14.43	16.30	17.73	18.85	17.19	16.93
NLM	30.52	35.26	29.51	31.48	32.59	36.71	34.85	32.67
LMMSE	10.76	12.47	10.79	11.25	12.74	14.53	13.40	12.38
UWT	13.27	14.89	12.12	13.76	15.10	18.76	15.32	14.17

Overall, the following work has been completed in this paper. A novel method for denoising Rician noise in MR images was proposed. The adaptive NLM method can independently select the optimal search window size for each pixel according to the noise variance that is estimated by the improved MAD estimator. The FRFCM can divide the membership degrees of MR images to preserve the details well. However, the FANLM method takes a long time to select the window size, and the steps are slightly cumbersome. All these issues can be subjects in future studies.

## Conclusions

As described in this paper, we proposed an improved method to suppress the Rician noise of MR images. We designed three window sizes, and the optimal size of search window is selected according to the estimated region characteristics. By combining the FRFCM method with the adaptive NLM algorithm, we proposed the FANLM method to denoise the MR images. Using the FANLM method, the image details can be well preserved with the noise mostly being removed. The experimental results show that the proposed algorithm FANLM can effectively remove the noise and is better than the NLM, LMMSE and UWT methods.

## Data Availability

The datasets1 used in this study are publicly available from the BrainWeb [26]. The datasets 2 are available from the corresponding author on reasonable request.

## Abbreviations

ADF: Anisotropic diffusion filter
FANLM: Fuzzy c-means and adaptive non-local means
FCM: Fuzzy c-means
FLICM: Local fuzzy clustering algorithm
FRFCM: Robust FCM algorithm
LMMSE: Linear minimum mean square error
MAD: Median absolute deviation
MRI: Magnetic resonance image
MSE: Mean square error
NLM: Non-local means
PCA: Principal component analysis
PSNR: Peak signal-to-noise ratio
SSIM: Structural similarity
UWT: Undecimated wavelet transform
